# A bibliometric and scientific knowledge-map study of the chimeric antigen receptor (CAR) natural killer (NK) cell-related research from 2010 to 2022

**DOI:** 10.3389/fimmu.2022.969196

**Published:** 2022-08-12

**Authors:** Juan Zhang, Peng Chen, Lele Miao

**Affiliations:** ^1^ Senior Department of Hematology, the Fifth Medical Center of PLA General Hospital, Beijing, China; ^2^ Chinese PLA Medical School Beijing, Beijing, China; ^3^ Department of General Surgery, Second Hospital of Lanzhou University, the Second Clinical Medical College of Lanzhou University, Lanzhou, China; ^4^ Key Laboratory of the Digestive System Tumors of Gansu Province, Lanzhou, China

**Keywords:** CAR-NK cells, CiteSpace, VOSviewer, bibliometric, knowledge-map

## Abstract

**Objectives:**

As emerging adoptive immunotherapy after CAR-T cell therapy, CAR-NK cell therapy has been developing rapidly in recent years. Presently, the research on CAR-NK cells has become a hotspot in the field of tumor immunotherapy.

**Methods:**

In this descriptive study, CtieSpace and VOSviewer were used to perform the bibliometric and scientific knowledge-map analysis of articles and reviews related to CAR-NK cells.

**Results:**

5371 authors from 715 institutions in 65 countries published 1028 papers about CAR-NK cells in 346 journals. The number of publications related to CAR-NK cells was increasing overall, especially from 2018 to 2021. The United States was in a leading position. The most active institution was Univ Texas, MD Anderson Cancer Center (USA). The journal with the most publications was *Frontiers in immunology*, and the most co-cited journal was *Blood*. The researcher with the most published papers was Winfried S. Wels, while the most co-cited researcher was Shannon L Maude. The research of CAR-NK cells in hematological malignancies and solid tumors (especially the selection of targets and the evaluation of efficacy and safety) was a research hotspot in this field. The emerging topics mainly included three aspects. First, further improve the proliferation and persistence of NK cells *in vivo*. Secondly, optimizing and improving the CAR structure for NK cells to improve the anti-tumor ability of CAR-NK cells. Thirdly, the related research of CRISPR/Cas9 gene-editing technology in constructing engineered immune cells.

**Conclusion:**

In this study, a bibliometric and scientific knowledge-map study provided a unique and objective perspective for the CAR-NK cell field. This information would provide a helpful reference for researchers interested in this field.

## Introduction

Chimeric antigen receptor (CAR)-T cell therapy has made a tremendous breakthrough in the research and treatment of hematological malignancies, bringing new hope to patients. Five CAR-T cell products have been approved by the U.S. Food and Drug Administration (FDA) for the treatment of hematological tumors. The success of CAR-T cell therapy aroused researchers’ interest in this adoptive immunotherapy. With the development of genetic modification technology, NK cells have been further customized, including introducing CAR and knocking out suppressor genes (1). Therefore, NK cells, which are thought to have the same potential to enhance the anti-tumor ability through CAR modification (2), gradually come into the researchers’ field of vision.

As innate immune effector cells, NK cells are essential members of the immune system and have broad-spectrum anti-tumor effects. NK cells can rapidly kill abnormal cells without pre-sensitization and MHC restriction (3). Moreover, NK cells play a killing role in four ways (4–7), including perforin/granzyme pathway, Fas/FasL pathway, cytokine pathway, and antibody-dependent cell-mediated cytotoxicity (ADCC) mediated by cluster of differentiation-16 (CD16).

Compared with CAR-T cells, CAR-NK cells have some disadvantages, including low persistence (8), proliferation *in vivo* only in the presence of specific cytokines (such as IL-2 and IL-15) (9), and lack effective CAR gene transfer methods (9). CAR-NK cells also have some advantages, including 1. There are many sources of NK cells (10–12), such as NK cell lines, peripheral blood, cord blood and induced pluripotent stem cells (iPSCs), especially NK92 cells (13–15); 2. Higher security. Graft-versus-host disease (GVHD) hardly occurs, and the incidence of cytokine release syndrome (CRS) and neurotoxicity is also very low (16); 3. In addition to the CAR-dependent mechanism, the anti-tumor mechanism of CAR-NK cells also includes a CAR-independent (NK cell receptor-dependent) mechanism (17–19). It is these advantages that make the research on CAR-NK cells become the research focus in the field of tumor immunotherapy (20, 21).

As a hot field, research and publications related to CAR-NK cells are increasing year by year. For those researchers who are initially involved in CAR-NK cell research, especially non-professionals interested in this field, it is difficult to quickly grasp and summarize the relevant knowledge in a short time. The bibliometric analysis can well summarize and analyze these numerous documents and complicated data. However, currently, there are no bibliometric studies on CAR-NK cells. This study is the first time to comprehensively and objectively analyze the evolution process, development trend, important knowledge, research hotspots and emerging topics in this field by combining bibliometrics analysis with knowledge-map analysis.

Bibliometrics analyzes literature quantitatively and objectively through mathematical and statistical methods. Through bibliometric analysis of literature on a particular topic, this field’s development and change trend and discover the research hotspots and the latest research topics can be understand quickly (22, 23). In addition, it can also help us obtain some critical information, including the contributions and cooperation of countries, institutions and authors, as well as the contributions and the topic distribution of journals. In this study, the bibliometric analysis of the CAR-NK cell field was carried out, and relevant scientific knowledge maps were constructed. It aimed to explore the research overview and development trend in the CAR-NK cell field and seek research hotspots and emerging topics. We hope this study can provide new clues and ideas for researchers in the CAR-NK cell field, and provide convenience and help for researchers who are initially involved in this field.

## Materials and methods

### Data collection

The relevant literature was retrieved and downloaded from WoSCC at 21:36 on May 2^nd^, 2022. The retrieval formula was shown in Annexes 1. 1028 papers were obtained, including 454 articles and 574 reviews (Annexes 2).

### Data analysis and visualization

As a citation visualization software, CtieSpace is good at mining and analyzing potential information in literature, including citation and co-citation times, subject distribution, contributions and cooperation of countries and institutions, contributions and cooperation of authors, research hotspots, and emerging topics (24). VOSviewer, a software for bibliometric mapping, has a strong capability of bibliometric network analysis and is better at constructing the visual network maps of scientific knowledge (25).

The acquired data was processed and visualized by Microsoft Office Excel 2010, CtieSpace (version 5.8.R3) and VOSviewer (version 1.6.17). All three researchers involved in this study were able to use the above software to process data proficiently. Two researchers (JZ and LM) independently analyzed and extracted the following information (1): the number of papers published each year; (2) contribution degree, centrality and cited times of countries/institutions; (3) contribution and citation times of authors; (4) contribution degree, impact factor and journal citation reports (JCR) region of journals; (5) Discipline distribution; (6) the frequency of keywords; and (7) co-citation times and centrality of references. Two people used Microsoft Excel to store and analyze data. Disagreements were resolved through discussion with the third reviewer (PC). In this descriptive study, variables were presented as numbers and percentages. No comparisons were made, therefore, no *P* values was set.

## Results

### The annual growth trend of publications

**Figure 1:**
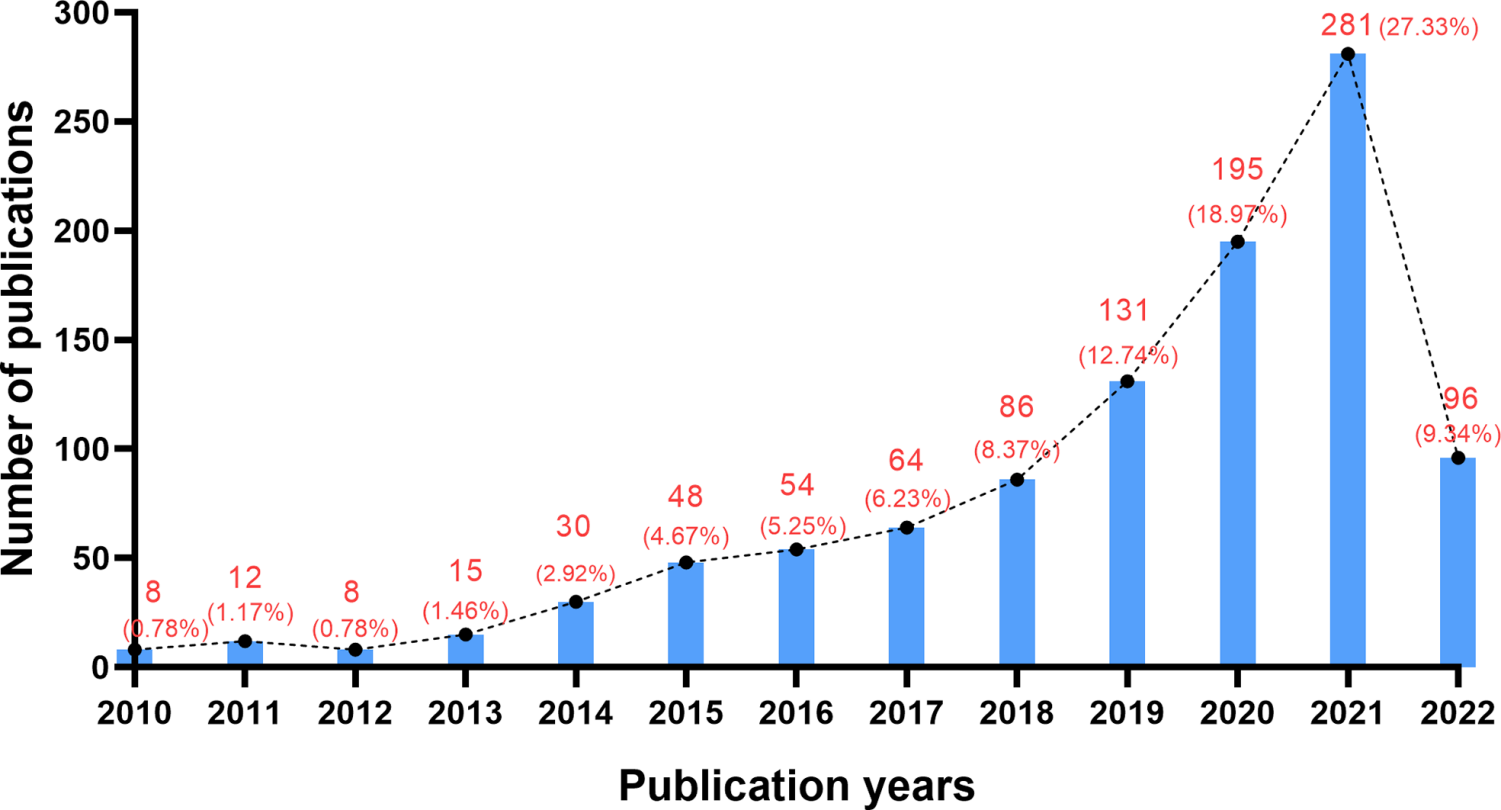
The trend of publication outputs about CAR-NK cells.

By counting the number of papers related to the CAR-NK cell field each year, we can know the development speed and trend of the field. According to the above retrieval strategy, 1028 papers about CAR-NK cells were obtained. **Figure 1** shows that the number of papers in this field was rising (2010-2021). This trend could be divided into the stagnation period (2010-2012), the slow growth period (2013-2017) and the rapid growth period (2018-2021). In the stagnation period, less than 15 papers were published each year. This situation lasted for about 3 years, with 57 papers (only 2.7% of the total). However, from 2018 to 2021, a total of 693 papers were published (accounting for 67.4% of the total), far exceeding the sum of the past eight years.

### Countries and institutions

**Figure 2:**
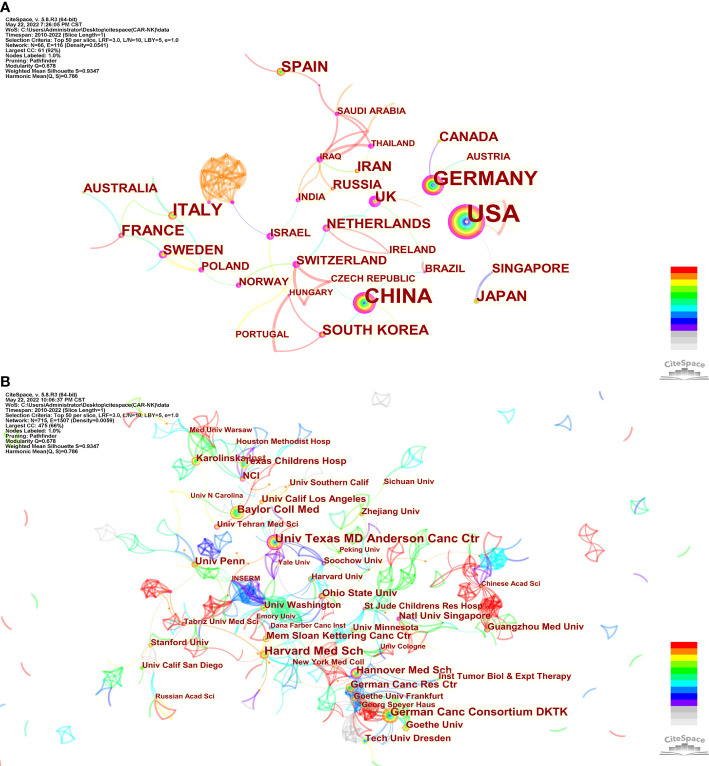
The co-occurrence map of countries **(A)** and institutions **(B)** in the CAR-NK cell field.

715 institutions from 65 countries co-authored 1028 papers (as an article might be completed by cooperation of several countries or organizations, it would be counted many times). **Table 1** shows that the USA published the most papers (n = 461, 33.50%) in the field related to CAR-NK cells, followed by China (n = 184, 13.37%) and Germany (n = 137, 9.96%), and only these three countries published more than 100 papers. The country with the most citations was still USA (n = 16250), with an average of 35.2 citations per paper. Moreover, among the top 10 countries, UK had the highest centrality (0.7), followed by Germany (0.37), the China (0.25) and the USA (0.22). It means that these countries, especially UK, have played an important role as a bridge in this field. Additionally, 90% of the top10 countries were developed countries. From **Figure 2A**, we can intuitively see the contribution of these countries to this field (the size of the circle), the cooperation between countries (the lines between the circles), and the countries with high centrality (with purple circles).

**Table 1:** The top 10 countries and institutions involved in CAR-NK cells.

Rank	country	Count	Centrality	Total Citations	Institution	Count	Centrality	Total Citations
1	USA	461 (33.50%)	0.22	16250	Univ Texas MD Anderson Canc Ctr (USA)	43 (2.53%)	0.28	2726
2	CHINA	184 (13.37%)	0.25	4929	Harvard Med Sch (USA)	37 (2.18%)	0.05	725
3	GERMANY	137 (9.96%)	0.37	4725	German Canc Consortium DKTK (Germany)	30 (1.76%)	0.02	1240
4	ITALY	67 (4.87%)	0.09	1029	Baylor Coll Med (USA)	26 (1.53%)	0.03	1590
5	SPAIN	38 (2.76%)	0.06	957	Hannover Med Sch (Germany)	24 (1.41%)	0.21	861
6	FRANCE	36 (2.62%)	0.13	1072	German Canc Res Ctr (Germany)	22 (1.29%)	0.07	755
7	JAPAN	34 (2.47%)	0	626	Univ Penn (USA)	21 (1.23%)	0.06	594
8	UK	32 (2.33%)	0.7	756	Mem Sloan Kettering Canc Ctr (USA)	20 (1.18%)	0.02	692
9	IRAN	29 (2.11%)	0	228	Ohio State Univ (USA)	19 (1.12%)	0.08	1092
10	SOUTH KOREA	26 (1.89%)	0.13	330	Goethe Univ (Germany)	18 (1.06%)	0	452

The institution with the most papers published was Univ Texas, MD Anderson Cancer Center (n = 43, 2.53%), followed by Harvard Med Sch (n = 37, 2.18%) and German Canc Consortium DKTK (n = 30, 1.76%). Furthermore, the institution with the most citations was still Univ Texas MD Anderson Cancctr (n = 2726), with an average of 63.4 citations per paper. Among the top10 institutions, 60% and 40% were from USA and Germany respectively (**Table 1**). **Figure 2B** shows intensive cooperation among institutions, and this cooperation is mainly concentrated from 2016 to 2021.

### Journals and co-cited journals

**Figure 3:**
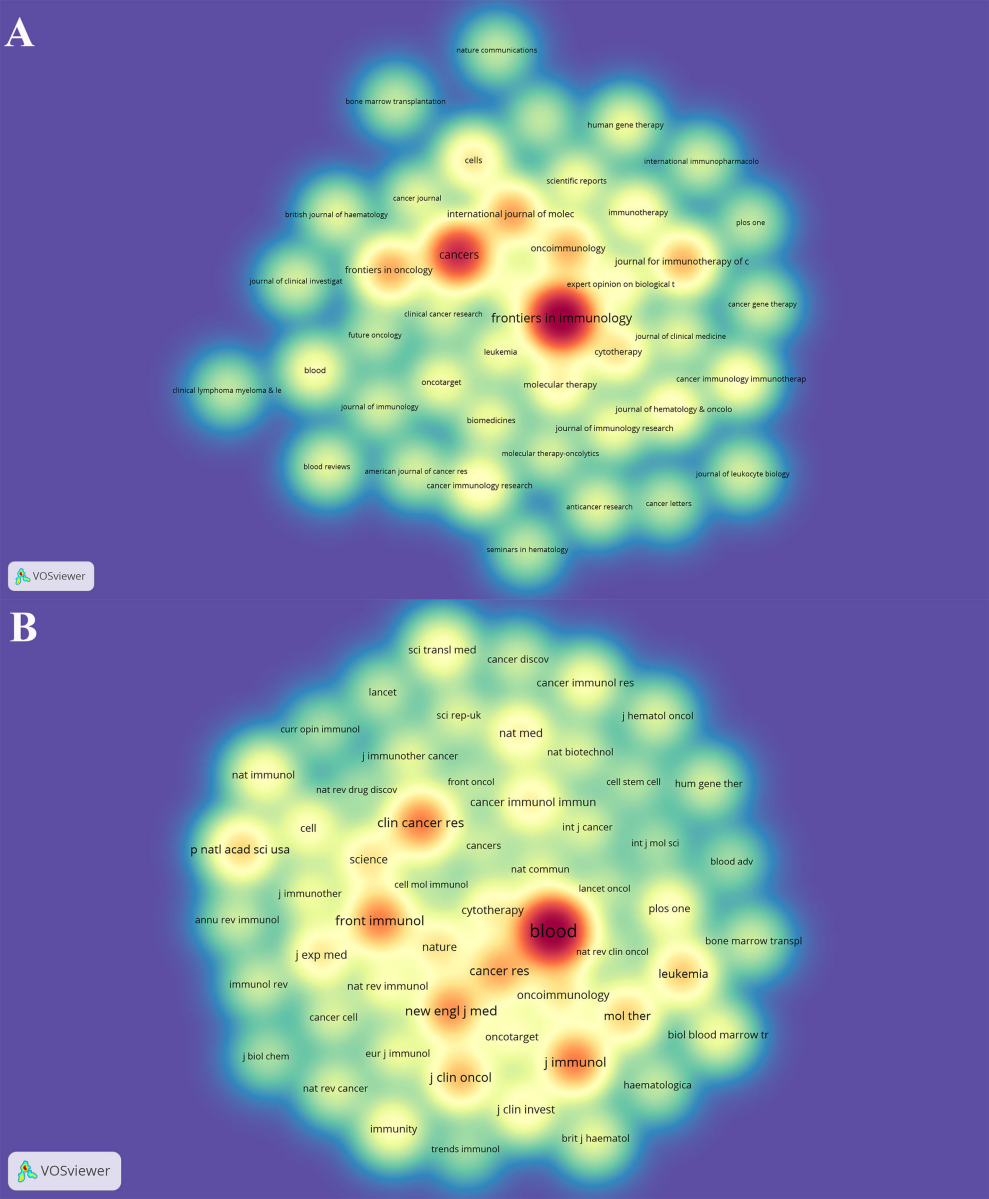
The density map of journals **(A)** and co-cited journals **(B)** in the CAR-NK cell field. Figure A shows journals with a number of publications ≥5; Figure B shows the journals with citations ≥100.

1028 CAR-NK cell-related publications were published in 346 academic journals. **Table 2** shows the top 10 journals with published papers and the top 10 journals with co-citations. The journal with the most publications was *Frontiers in Immunology* (n = 95, 9.24%), followed by *Cancers* (n = 60, 5.84%) and *International Journal of Molecular Sciences* (n = 27, 2.63%). There were 6 journals with at least 20 publications. **Figure 3A** can intuitively display those journals with more publications (the darker the color, the more publications).

**Table 2:** The top 10 journals and co-cited journals related to CAR-NK cells.

Rank	Journal	Counts	IF(2020)	JCR(2020)	Co-cited journal	Coitations	IF(2020)	JCR(2020)
1	Frontiers in Immunology	95 (9.24%)	7.561	Q2	Blood	9612 (8.91%)	23.629	Q1
2	Cancers	60 (5.84%)	6.639	Q2	Journal of Immunology	3389 (3.14%)	5.422	Q1
3	International Journal of Molecular Sciences	27 (2.63%)	5.924	Q3	Clinical Cancer Research	3346 (3.10%)	12.531	Q1
4	Frontiers in Oncology	26 (2.53%)	6.244	Q2	Frontiers in immunology	3325 (3.08%)	7.561	Q2
5	Oncoimmunology	24 (2.33%)	8.110	Q1	New England Journal of Medicine	2963 (2.75%)	91.253	Q1
6	Journal for Immunotherapy of Cancer	24 (2.33%)	13.751	Q1	Cancer Research	2594 (2.41%)	12.701	Q1
7	Cytotherapy	17 (1.65%)	5.414	Q1	Journal Clinical Oncology	2320 (2.15%)	44.544	Q1
8	Cells	16 (1.56%)	6.600	Q3	leukemia	2082 (1.93%)	11.528	Q1
9	molecular therapy	14 (1.36%)	11.454	Q1	molecular therapy	1976 (1.83%)	11.454	Q1
10	Immunotherapy cancer immunology research journal of hematology & oncology	13 (1.26%) 13 (1.26%) 13 (1.26%)	4.196 11.151 17.388	Q3 Q1 Q1	Proceedings of the National Academy of Sciences of the United States of America	1948 (1.81%)	11.205	Q1

IF: Impact Factor. JCR: Journal citation reports.

It can also be seen from **Table 2** that *Blood* (n = 9612, 8.91%) is the most co-cited journal, far exceeding other journals, followed by *Journal of Immunology* (n = 3389, 3.14%) and *Clinical Cancer Research* (n = 3346, 3.10%). Furthermore, 10 journals were co-cited more than 1,900 times, and 8 journals had an impact factor (IF) more than 10. **Figure 3B** intuitively displays those journals with more co-citations (the darker the color, the more co-citations).

**Figure 4:**
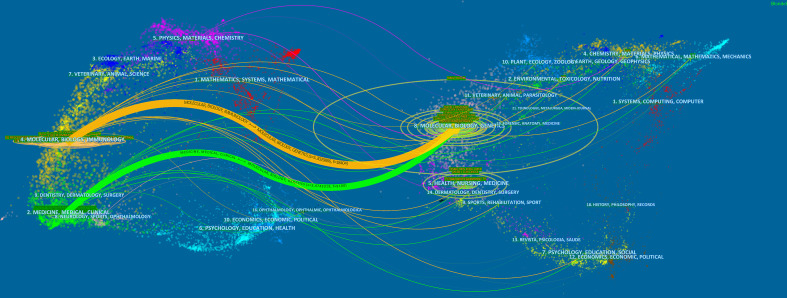
The dual-map overlay of journals on CAR-NK cells. Image parameter: a: 3; Source Circle Size: 200; Target Circle Size: 8; Snap to centroids (< Radius): 0.

**Figure 4** shows the distribution of journal topics and the citation relationship between journals. Additionally, there were two main reference paths (one orange and one green) in it. It suggests that the papers published in “Molecular/Biology/Genetics” journals were often cited by the papers published in “Molecular/Biology/Immunology” journals and “Medicine/Medical/Clinical” journals.

### Authors and co-cited authors

**Figure 5:**
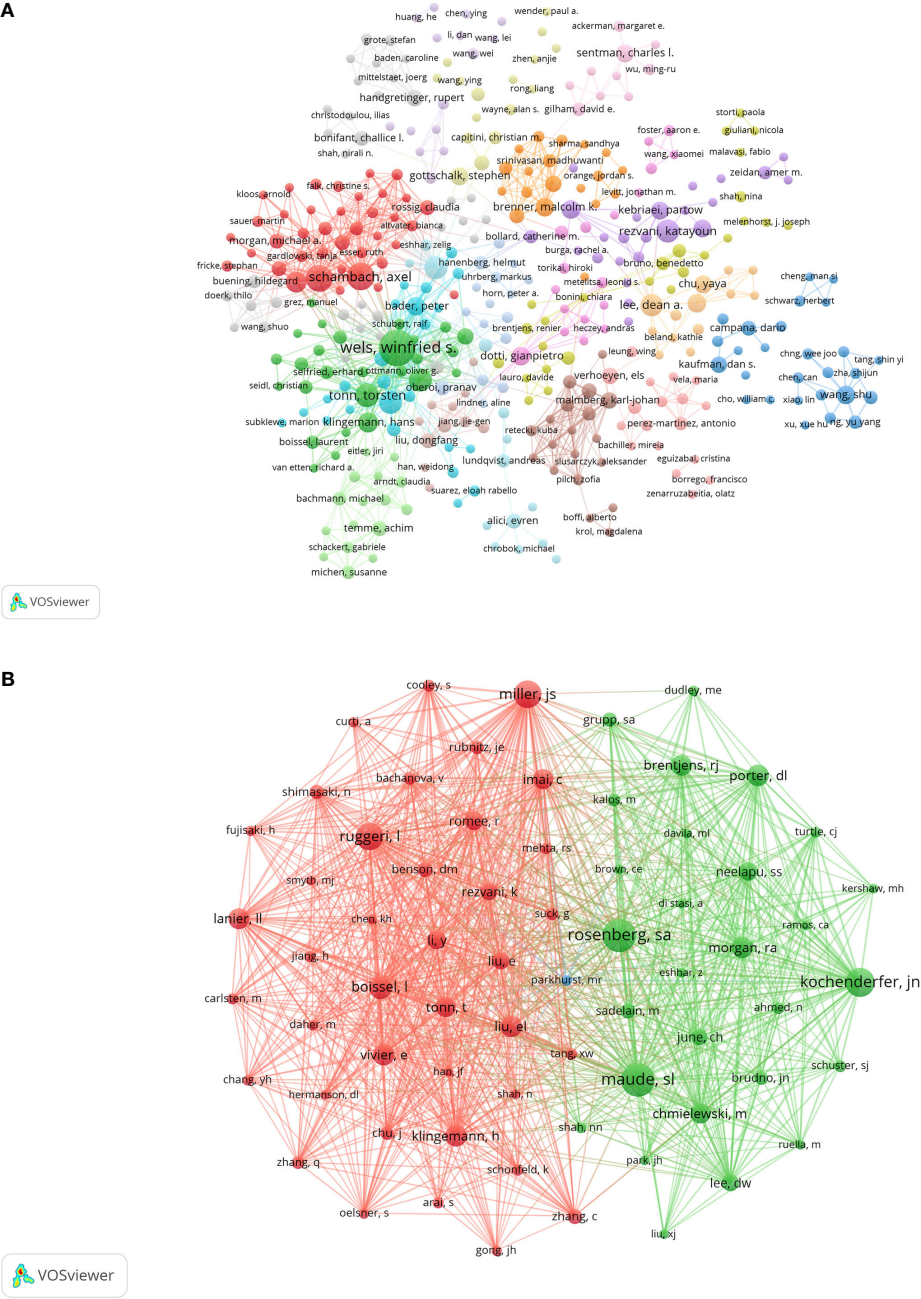
The visualization map of authors **(A)** co-cited authors **(B)** involved in CAR-NK cells. Minimum number of documents of an aduthor ≥2; Note: Minimum number of citations of an aduthor ≥100.

A total of 5,371 researchers participated in the publication of these papers. The top 10 researchers had published at least 10 papers (**Table 3**). Winfried S. Wels (n = 30) published the most papers, followed by Axel Schambach (n = 17), Ulrike Koehl (n = 16) and Torsten Tonn (n = 13). We screened authors who had published at least 2 papers to construct the corresponding collaboration network map (**Figure 5A**). It shows 18 clusters of different colors, and the authors in the same cluster have active cooperation.

**Table 3:** The top 10 authors and co-cited authors of CAR-NK cell researches.

Rank	Author	Counts	Citations	Rank	Co-cited author	Citations
1	Winfried S. Wels	30	2127	1	Maude SL	365
2	Axel Schambach	17	475	2	Rosenberg SA	362
3	Ulrike Koehl	16	811	3	Kochenderfer JN	313
4	Torsten Tonn	13	1280	4	Miller JS	301
5	Hinrich Abken	12	487	5	Ruggeri L	299
6	Katayoun Rezvani	12	790	6	Boissel L	240
7	Evelyn Ullrich	12	349	7	Klingemann H	235
8	Congcong Zhang	12	911	8	Vivier E	232
9	Dean a. Lee	11	521	9	Liu EL	231
10	Stephan Kloess	10	640	10	Morgan RA Porter DL	229 229

68 authors co-cited more than 100 times in this study. The most frequently co-cited researchers were Maude SL (n = 365), followed by Rosenberg SA (n = 362), Kochendorfer JN (n = 313) and Miller JS (n = 301). These 68 authors were included to construct a co-citation network map (**Figure 5B**). **Figure 5B** shows these co-cited authors and co-cited relationships more intuitively.

### Co-occurrence, clusters, and evolution of keywords

**Figure 6:**
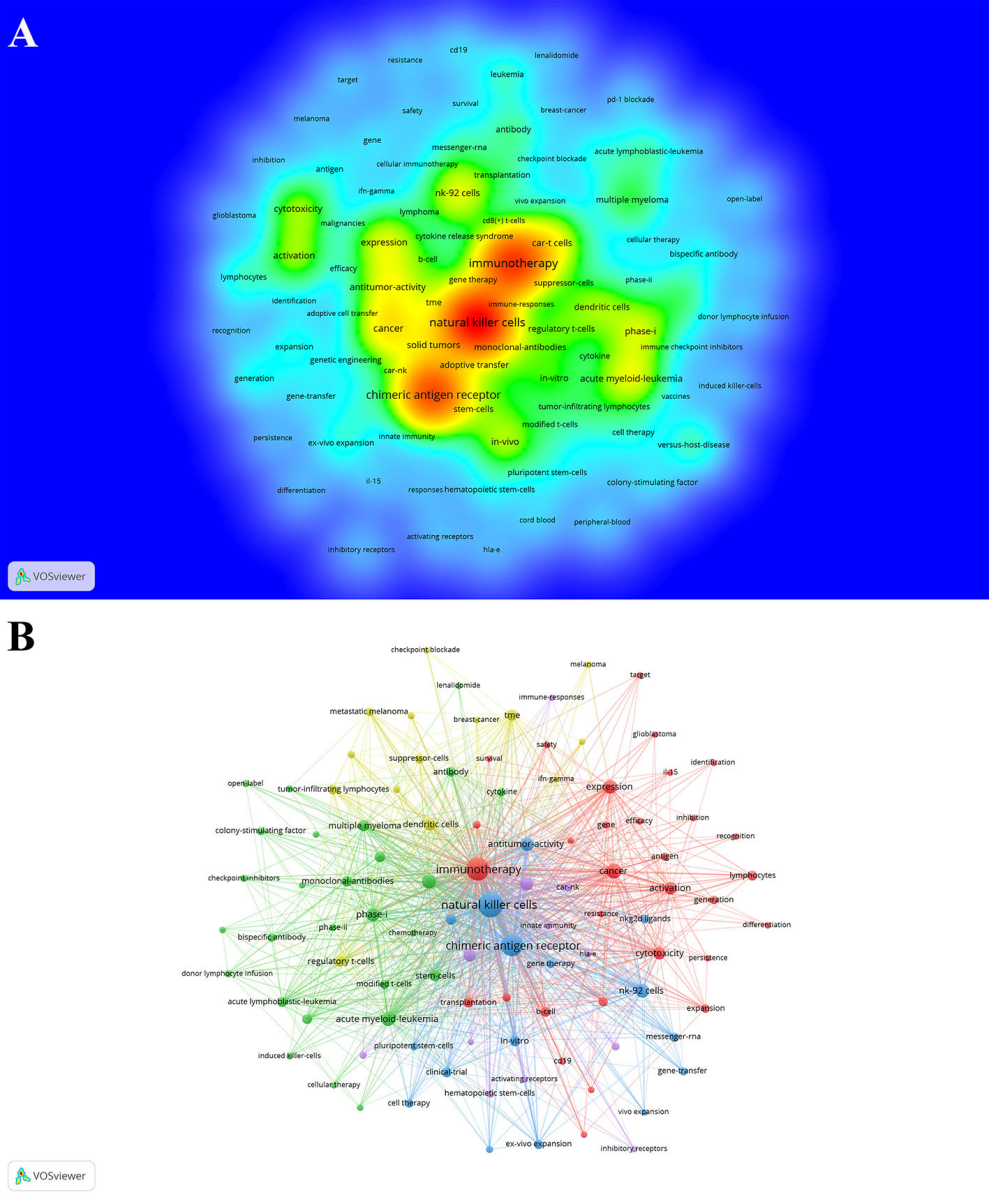
The co-occurrence density map **(A)** and network **(B)** of keywords involved in CAR-NK cells. Minimum number of occurrences of keywords ≥15.

Keywords generally reflect the theme and research content of a paper. We can rapidly understand one certain field’s research focus and direction through the co-occurrence analysis of keywords. After merging some duplicate keywords, a total of 3908 keywords were obtained. **Table 4** shows the top 20 keywords. The most frequently occurring keywords were natural killer cells (n = 735), followed by immunotherapy (n = 574), chimeric antigen receptor (n = 504), and cancer (n = 178). A total of 100 keywords with at least 15 occurrences were included, and a density map of keywords (**Figure 6A**) was constructed with these keywords. Those high-frequency keywords can be found more easily in **Figure 6A**.

**Table 4:** Top 20 keywords related to CAR-NK cells.

Rank	Keyword	Count	Rank	Keyword	Count
1	natural killer cells	735	11	solid tumors	117
2	immunotherapy	574	12	in-vivo	110
3	chimeric antigen receptor	504	13	cytotoxicity	108
4	cancer	178	14	activation	107
5	car-t cells	143	15	dendritic cells	96
6	acute myeloid-leukemia	141	16	tme	78
7	nk-92 cells	138	17	multiple myeloma	77
8	expression	132	18	regulatory t-cells	72
9	antitumor-activity	125	19	stem-cells	70
10	phase-i	123	20	monoclonal-antibodies	67

Keywords with at least 15 occurrences were included, and a network cluster analysis of these keywords (**Figure 6B**) was constructed. A total of 5 different clusters were obtained. There were 31 keywords in cluster 1 (red), including immunotherapy, cancer, leukemia, glioblastoma, efficacy, resistance, CRS, cytotoxicity, and safety. There were 27 keywords in cluster 2 (green), including CAR-T cells, acute myeloid-leukemia(AML), acute lymphoblastic-leukemia(ALL), multiple myeloma (MM), phase-I, phase-II. There were 16 keywords in cluster 3 (blue), including CAR, NK cells, antitumor-activity, in-vitro, clinical-trial, NKG2D ligands, NK-92 cells, cord blood, and pluripotent stem-cells. There were 14 keywords in cluster 4 (yellow), including dendritic cells(DC), CD8+ T cells, regulatory T-cells, suppressor-cells, tumor-associated macrophages, tumor-infiltrating lymphocytes, and TME. There were 12 keywords in cluster 5 (purple), including CAR-NK, solid tumors, activating receptors, inhibitory receptors, and immune-responses.

**Figure 7:**
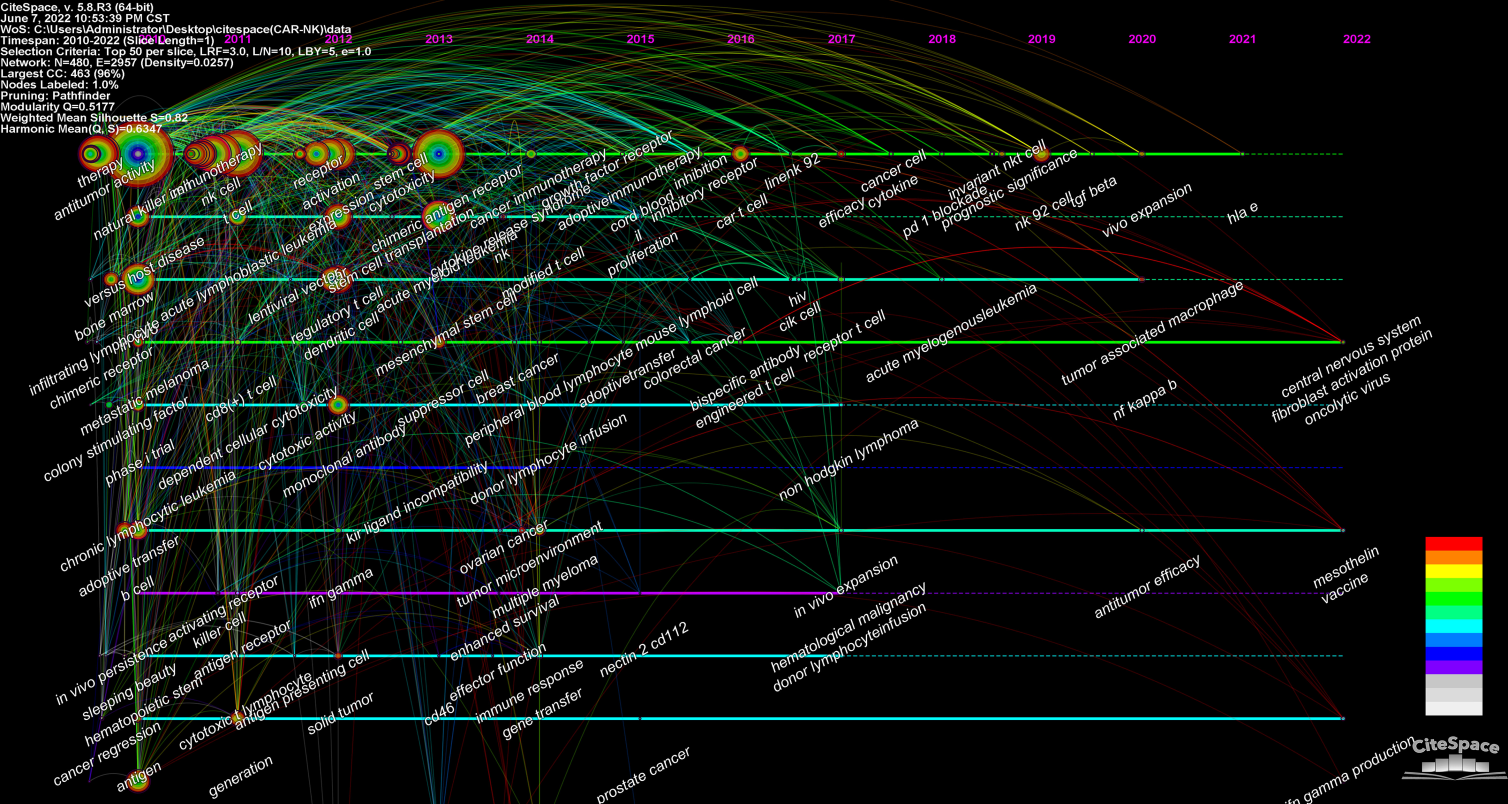
The timeline viewer of keywords involved in CAR-NK cells.

The Timeline viewer of keywords adds a time factor to the clustering of these keywords. It can help us explore the evolution track of these keywords in different topics. From **Figure 7**, we can intuitively see the evolution track of keywords in this field and focused keywords in each stage.

### Co-cited references and reference burst

**Table 5** consisted of two parts. The first part showed the top 10 co-cited articles about the field. The publication time was from 2014 to 2020. The top 3 articles were co-cited more than 150 times. The co-cited article the most co-citations was “Use of CAR-transferred natural killer cells in CD19-positive lymphoid tumors” (n = 214), published by Liu et al. (16) in 2020. The second part showed the top 10 co-cited reviews related to CAR-NK cells. The publication time was from 2015 to 2020. The most frequently co-cited review was “Engineering Natural Killer Cells for Cancer Immunotherapy” (n = 89) by Rezvani et al. (34) in 2017.

**Table 5:** The top 10 co-cited references related to CAR-NK cells. .

Top 10 co-cited references (only including articles) related to CAR-NK cells						
Rank	Year	Author	Title	Journal	Co-citation	Centrality
1	2020	Liu EL et al. (16)	Use of CAR-Transduced Natural Killer Cells in CD19-Positive Lymphoid Tumors	N Engl J Med	214	0.01
2	2018	Liu EL et al. (26)	Cord blood NK cells engineered to express IL-15 and a CD19-targeted CAR show long-term persistence and potent anti-tumor activity	Leukemia	173	0.04
3	2018	Li Y et al. (27)	Human iPSC-Derived Natural Killer Cells Engineered with Chimeric Antigen Receptors Enhance Anti-tumor Activity	Cell Stem Cell	153	0.01
4	2018	Tang XW et al. (28)	First-in-man clinical trial of CAR NK-92 cells: safety test of CD33-CAR NK-92 cells in patients with relapsed and refractory acute myeloid leukemia	Am J Cancer Res	112	0.01
5	2017	Zhang CC et al. (20)	Chimeric Antigen Receptor-Engineered NK-92 Cells: An Off-the-Shelf Cellular Therapeutic for Targeted Elimination of Cancer Cells and Induction of Protective Antitumor Immunity	Front Immunol	87	0.01
6	2015	Schönfeld K et al. (29)	Selective inhibition of tumor growth by clonal NK cells expressing an ErbB2/HER2-specific chimeric antigen receptor	Mol Ther	86	0.01
7	2014	Chu J et al. (30)	CS1-specific chimeric antigen receptor (CAR)-engineered natural killer cells enhance *in vitro* and *in vivo* antitumor activity against human multiple myeloma	Leukemia	83	0.02
8	2016	Romanski A et al. (31)	CD19-CAR engineered NK-92 cells are sufficient to overcome NK cell resistance in B-cell malignancies	J Cell Mol Med	70	0.01
9	2015	Zhang CC et al. (32)	ErbB2/HER2-Specific NK Cells for Targeted Therapy of Glioblastoma	J Natl Cancer Inst	64	0.01
10	2017	Oelsner S et al. (33)	Continuously expanding CAR NK-92 cells display selective cytotoxicity against B-cell leukemia and lymphoma	Cytotherapy	63	0
Top 10 co-cited references (only including reviews) related to CAR-NK cells						
1	2017	Rezvani K et al. (34)	Engineering Natural Killer Cells for Cancer Immunotherapy	Mol Ther	89	0
2	2016	Suck G et al. (35)	NK-92: an ‘off-the-shelf therapeutic’ for adoptive natural killer cell-based cancer immunotherapy	Cancer Immunol Immunother	84	0.01
3	2016	Klingemann H et al. (36)	Natural Killer Cells for Immunotherapy - Advantages of the NK-92 Cell Line over Blood NK Cells	Front Immunol	80	0
4	2018	Mehta RS et al. (37)	Chimeric Antigen Receptor Expressing Natural Killer Cells for the Immunotherapy of Cancer	Front Immunol	75	0
5	2015	Glienke W et al. (38)	Advantages and applications of CAR-expressing natural killer cells	Front Pharmacol	63	0.01
6	2020	Xie GZ et al. (39)	CAR-NK cells: A promising cellular immunotherapy for cancer	EBioMedicine	33	0
7	2016	Guillerey C et al. (40)	Targeting natural killer cells in cancer immunotherapy	Nat Immunol	49	0
8	2018	Hu Y et al. (41)	Chimeric antigen receptor (CAR)-transduced natural killer cells in tumor immunotherapy	Acta Pharmacol Sin	49	0
9	2019	Hu WL et al. (42)	Cancer Immunotherapy Based on Natural Killer Cells: Current Progress and New Opportunities	Front Immunol	35	0
10	2015	Rezvani K et al. (43)	The Application of Natural Killer Cell Immunotherapy for the Treatment of Cancer	Front Immunol	29	0

**Figure 8:**
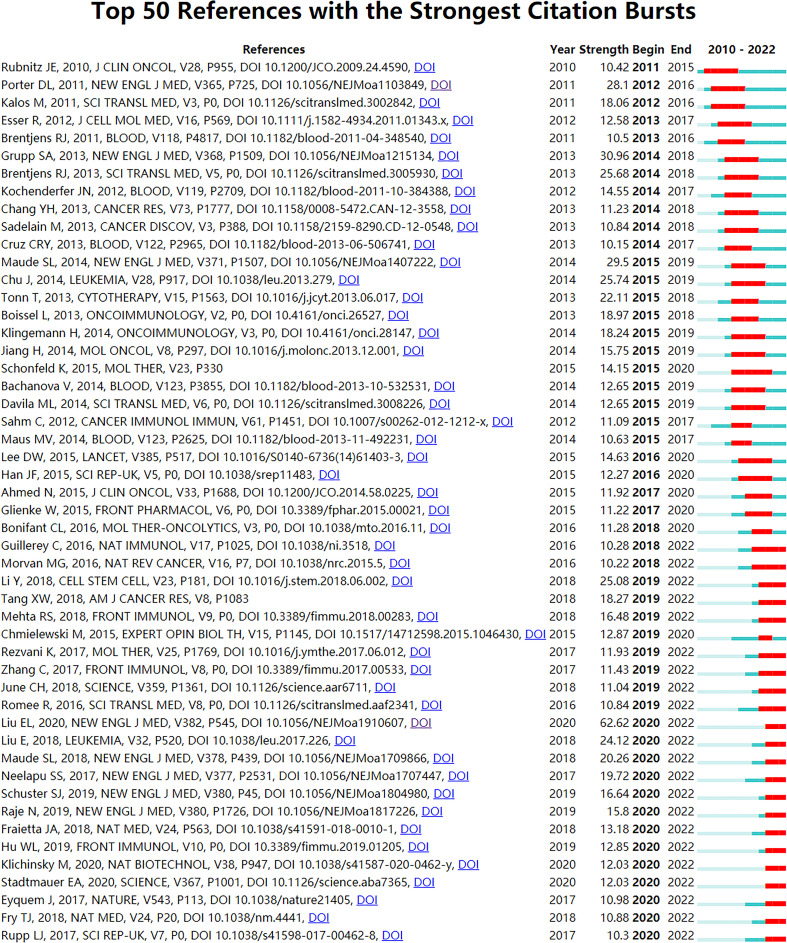
The top 50 references with the strongest citation bursts involved in CAR-NK cells. The Blue bars mean the reference had been published; the red bars mean citation burstness.

References with citation bursts refer to references whose citations suddenly increase in a certain period. 255 references with the strongest citation bursts were obtained through CiteSpace (selection criteria: top 100 per slice; minimum duration: 2). We chose the top 50 (**Figure 8**). The reference with the strongest burstness (strength = 62.62) was “Use of CAR-Transduced Natural Killer Cells in CD19-Positive Lymphoid Tumors (16)” (publication year: 2020). Among these 50 references, 46 were published from 2012 to 2022, and 19 (38%) were published from 2017 to 2022. It indicates that these papers were frequently cited within 10 years and 5 years. More importantly, 22 (44%) of these 50 papers are currently in citation burstness. All these mean that the research about CAR-NK cells will continue to receive attention in the future. Furthermore, 10 of the 22 papers were related to CAR-T cells, which indicates that the CAR-T cell field has a significant influence on the research field of CAR-NK cells.

## Discussion

CAR-T cell therapies have made breakthroughs in the treatment of hematological malignancies, and five CAR-T cell therapies have been approved for marketing. However, there is still no CAR-T cell therapy for solid tumors on the market. This is because, compared with hematological malignancies, CAR-T cells face greater limitations in solid tumors, such as the homing obstacle of CAR-T cells and the inhibition of tumor microenvironment (TME). As a new anti-tumor immunotherapy, CAR-NK cell therapy has great potential. Compared with CAR-T cells, the sources of NK cells used to construct CAR-NK cells are extremely wide (10–12), CAR-NK does not cause GVHD, and the incidence of CRS and neurotoxicity is extremely low (16). Moreover, NK cells themselves have multiple anti-tumor mechanisms (17–19).

So far, over 30 registered CAR-NK cell-related clinical trials have been retrieved (clinicaltrials.gov). Although CAR-NK cell therapy has some unique advantages, it also has some limitations that cannot be ignored. These limitations are similar to those of CAR-T cell therapy, including lack of specific target antigens, antigen heterogeneity and challenges after infusion (44). Furthermore, CAR-NK cells also face many obstacles in solid tumors, such as homing obstacle of CAR-NK cells and inhibition of TME. Thus, there are still many problems and obstacles to be solved and broken through in this field.

In recent years, the field of CAR-NK cells has been developing rapidly, and new knowledge has been generated like a blowout. It is very difficult to quickly grasp the core knowledge in a huge knowledge base. The bibliometric analysis can well summarize and analyze these numerous documents and complicated data. This study obtained some valuable results in the CAR-NK cell field through bibliometric analysis and knowledge-map analysis, including contributions and cooperation of countries/institutions/authors, contributions and topic distribution of journals, development trends, important knowledge, research hotspots, and emerging topics. In this study, we expounded and discussed the general information, knowledge base, hotspot evolution, emerging topics related to CAR-NK cells, and the limitations of our research.

### General information

The number of papers was on the rise, which was divided into the stagnation period (2010-2012), the slow growth period (2013-2017) and the rapid growth period (2018-2021) (**Figure 1**). In the stagnant period, the research on CAR-NK cells was inactive, and the number of papers published each year was less than 5. During the slow growth period, the number of CAR-NK cell-related papers increased slowly, indicating that this field was attracting attention. Interestingly, there was an obvious turning point in this stage; after 2012, the number of publications has increased substantially. This phenomenon may be related to an important event. Emily Whitehead, 7-year-old with ALL, achieved complete remission after receiving CD19-CAR-T cell therapy (45). This event made CAR-T cells begin to attract widespread attention. The progress of CAR-T cell research has also promoted research in the field of CAR-NK cells (20). In the period of rapid growth, the field has developed rapidly, which indicates that the research on CAR-NK cells has aroused great interest of researchers. It should be mentioned that 693 papers (accounting for 67.4% of the total) were published in this stage, exceeding the sum of the past 8 years. According to the current trends, the number of publications in this field may continue to show positive growth.

The USA (n = 461, accounting for 33.50% of the total) published the most papers in the CAR-NK cell field, far exceeding other countries. UK had the highest centrality (0.7), which means that UK played an important role as a bridge in this field. Besides, 90% of the top10 countries were developed countries. It may be because the research and development of CAR-NK cells need much financial support. Among the top10 institutions, 60% and 40% were from USA and Germany respectively. The institution with the most papers published the most citations was Univ Texas, MD Anderson Cancer Center from the USA. Besides, **Figure 2B** shows intensive cooperation among institutions, and this cooperation is mainly concentrated from 2016 to 2021.

*Frontiers in Immunology* published the most papers (n = 95, 9.24%), while *Blood* was the most co-cited journal (n = 9612, 8.91%). **Figure 4** suggests that the research on CAR-NK cells mainly focuses on two aspects, including basic studies and translational medicine.

Through the authors and co-cited authors analysis, we could find the authors with the most published papers and the most co-cited authors in the CAR-NK cell field. In our analysis, Winfried S. Wels published the most papers (n = 30), while Shannon L Maude had the most co-citations (n = 365). Moreover, there was intensive cooperation among researchers in the same cluster and active cooperation among different clusters (**Figures 5A**).

The number of publications related to CAR-NK cells is increasing year by year, especially in recent years. As a hot field, CAR-NK cell-related research is attracting more and more researchers. By reading the relevant papers of experts (including those listed in **Table 3**) in this field, it will undoubtedly help readers to understand and master the research status and focus of the CAR-NK cell field. From the general information, we also obtain some important information, including: 1. In the field of CAR-NK cells, the United States is in an absolute leading position; 2. Journals in the field of tumor and immunity are interested in CAR-NK cell-related research. Overall, the general information can help readers quickly understand the general situation of this field.

### Knowledge base

The knowledge base is a collection of co-cited references (46). The 10 most co-cited papers in CAR-NK cell-related fields (the first part of **Table 5**) were as follows:

The most co-cited paper (n = 159) was “Use of CAR-transduced natural killer cells in CD19-positive lymphoid tumors” by Liu et al. (16) in 2020. In this phase 1 and 2 trial, 11 patients with CD19+ non-Hodgkin lymphoma or CLL received CD19-CAR-NK cells (derived from cord blood). The experimental results showed that about 73% of patients responded to the treatment with high safety, and no CRS or ICANS appeared in all patients. The second-ranked paper was published in 2018 (26). This study was a preclinical experiment of the previous study. The third-ranked paper was published in 2018 (27). Researchers used human iPSC-derived NK cells to construct CAR-NK cells targeting mesothelin to treat ovarian cancer. The CAR consisted of the natural killer group 2 member D (NKG2D) transmembrane domain, 2B4 costimulatory domain and CD3ζ signaling domain (2nd generation CAR, 2B4). The results showed that mesothelin-CAR-NK cells exhibited similar anti-tumor activity to CAR-T cells *in vivo*, and the toxicity was lower. The fourth-ranked paper was published in 2018 (28). It was the first human clinical trial of CAR NK-92 cells (phase 1). 3 patients with RR-AML received CD33-CAR NK-92 cells (3rd generation CAR; CD28 and 4-1BB). The experimental results showed that although CD33-CAR NK-92 cells did not show apparent clinical efficacy in RR-AML, the experiment proved that this therapy was safe in these patients. The fifth-ranked paper was published in 2017 (20). Researchers introduced the research progress of HER2-CAR NK -92 cells in treating of HER2+ malignant tumors. Combined with their research, they proved that HER-CAR NK -92 cells (2nd generation CAR, CD28) could effectively control the tumor progression in the mouse models of HER2+ glioblastoma. The sixth-ranked paper was published in 2015 (29). This study showed that HER2-CAR NK-92 cells (2nd generation CAR, CD28) could recognize and kill HER2+ tumor cells. The seventh -ranked paper was published in 2014 (30). This study showed that CS1-CAR NK-92 cells (2nd generation CAR, CD28) could effectively inhibit tumor growth in the multiple myeloma (MM) xenograft mouse models. The eighth-ranked paper was published in 2016 (31). *In vitro* experiments showed that CD19-CAR NK -92 cells (1st generation CAR) could specifically and effectively kill CD19+ B-ALL cells. The ninth-ranked paper was published in 2015 (32). The research showed that in the mouse models, HER2-CAR NK -92 cells (2nd generation CAR, CD28) could specifically recognize and kill HER2+ glioblastoma cells and induce endogenous anti-tumor immunity. The tenth-ranked paper was published in 2017 (31). Researchers constructed three types of CD19-CAR NK-92 cells for the study of B-cell leukemia and lymphoma, including NK-92/63.z cells (1st generation CAR), NK-92/63.28.z cells (2nd CAR, CD28) and NK-92/63.137.z cells (2nd CAR, CD137). The study showed that all three CAR-NK cells could kill CD19+ B-cell leukemia and lymphoma cells, and the first two CAR-NK cells were more potent than the third one in cell killing and cytokine production.

Generally speaking, from these 10 most co-cited articles, we can obtain some vital information about CAR-NK cells, as follows:

- Currently, most research about CAR-NK cells is still in the basic research stage;
- CAR-NK cells are mainly used in the research about hematological malignancies and solid tumors, including anti-tumor effects and safety;
- The hematological malignancies and targets involved in these studies include non-Hodgkin lymphoma (CD19), CLL(CD19), ALL(CD19), AML(CD33) and MM (CS1);
- The solid tumors and targets involved in these studies mainly include glioblastoma (HER2), breast cancer (HER2) and ovarian cancer (mesothelin), especially glioblastoma. Beyond that, some recent studies on CAR-NK cells in solid tumors, such as lung cancer (DLL3) (47), gastric cancer (mesothelin) (48), pancreatic cancer (PSCA) (49), and prostate cancer (PSMA) (50).
- The main source for constructing CAR-NK cells is NK92 cells. It is mainly because 1. NK92 cells can expand indefinitely *in vitro* (51); 2. Reduced manufacturing time and cost of CAR-NK cells (39); 3. Reduced the sensitivity to freeze-thaw cycles (8). However, NK92 cells also have inherent disadvantages, including the risk of tumorigenesis, lack of CD16 (inability to trigger ADCC) (52), and requiring irradiation prior to infusion (loss of *in vivo* proliferative capacity) (29, 52).

The second part of **Table 5** is the 10 most co-cited reviews in the CAR-NK cell-related field. These reviews introduce and summarize the CAR-NK cell field from many aspects, which can help researchers understand the general situation, research focus and development trend.

Like CAR-T cell therapy, CAR-NK cell therapy also belongs to clinical application research, and its ultimate goal is to be used for clinical treatment. Up to now, only one clinical trial article (16) related to CAR-NK cells can be retrieved from Pubmed. Most research on CAR-NK cells is still in basic research and preclinical research stage. CAR-NK cells are mainly used in the research of hematological malignancies and solid tumors (9, 53, 54), and their anti-tumor effect and safety are the focus of attention, because these two aspects can affect the clinical promotion of this therapy. Additionally, because of some advantages of NK92 cells, they have become the main source of constructing CAR-NK cells, and have been approved for clinical applications (55).

### The analysis of hotspots and emerging topics

**Figure 7** shows the evolutionary trajectories of keywords and the topics focused in each stage in CAR-NK cell-related fields. During the stasis period (2010-2012), the keywords in this stage indicated that the relevant research mainly focused on the basic research or phase 1 clinical research (including the evaluation of efficacy, toxicity reaction and safety) of CAR-NK cells in hematological malignancies (mainly) and solid tumors. During the slow growth period (2013-2017), the research of CAR-NK cells in solid tumors gradually increased, for example, breast cancer, ovarian cancer, prostate cancer and colorectal cancer. In the period of rapid growth (2018-2021), it was the main research topic to explore further some intrinsic mechanisms (including immune checkpoints, TGF-β signaling pathway, NF-kB signaling pathway, and tumor-associated macrophages) to improve the antitumor efficacy of CAR-NK cells.

Keywords can generally reflect the theme and research content of a paper. **Table 4** shows the top 20 keywords. Some critical information in this field can be summarized through these keywords: 1. CAR-NK cell therapy is anti-tumor immunotherapy; 2. It is currently mainly used to study hematological malignancies (9, 56–58)and solid tumors (54, 59); 3. The anti-tumor effect of this therapy is the focus of attention; 4. Presently, CAR-NK cell research is mainly in basic research, while clinical trials are primarily in phase 1.

By summarizing the keywords of each cluster (**Figure 6B**), we can understand the scope and direction of research in this field. Cluster 1 keywords are mainly related to the research of immunotherapy in malignant tumors, including efficacy, safety and toxicity. Cluster 2 keywords are mainly related research of CAR-T cells in hematological malignancies. Cluster 3 keywords may be about the research of CAR-NK cells in tumors, and NK cells used in the experimental research are mostly derived from NK92 cells, pluripotent stem-cells and cord blood. Cluster 4 keywords are mainly related to tumor microenvironment. Cluster 5 keywords may be about the basic research of CAR-NK cells in solid tumors.

We can find emerging topics in a field by analyzing those references with citation bursts (60). Of these 50 papers, 22 (44%) were in the state of citation burstness (**Figure 8**). It suggests that the research about CAR-NK cells will continue to receive attention in the future. From these 22 papers, we screened 14 papers (9 articles and 5 reviews) related to CAR-NK cells or NK cells and arranged them according to their strength (**Table 6**). These 9 articles may represent emerging topics in the field. **Table 6** and **Table 5** show that 5 articles appear in both tables at the same time. These 5 articles (16, 20, 26–28) may be crucial references in CAR-NK cells (already introduced in the knowledge base). The remaining 4 articles in **Table 6** are introduced as follows:

**Table 6:** The references (in the state of citation burstness) related to CAR-NK cells or NK cells. .

NO.	Year	Author	Article Type	Targets	Associated Tumor	Title	Strength
1	2020	Liu E et al. (16)	article	CD19	Hodgkin’s lymphoma, chronic lymphocytic leukemia (CLL)	Use of CAR-Transduced Natural Killer Cells in CD19-Positive Lymphoid Tumors	60.9
2	2018	Li Y et al. (27)	article	mesothelin	ovarian cancer	Human iPSC-Derived Natural Killer Cells Engineered with Chimeric Antigen Receptors Enhance Anti-tumor Activity	25.08
3	2018	Liu E et al. (26)	article	CD19	hematological malignancies	Cord blood NK cells engineered to express IL-15 and a CD19-targeted CAR show long-term persistence and potent antitumor activity	24.12
4	2018	Tang XW et al. (28)	article	CD33	acute myeloid leukemia (AML)	First-in-man clinical trial of CAR NK-92 cells: safety test of CD33-CAR NK-92 cells in patients with relapsed and refractory acute myeloid leukemia	18.27
5	2018	Mehta RS et al. (37)	review			Chimeric Antigen Receptor Expressing Natural Killer Cells for the Immunotherapy of Cancer	16.48
6	2019	Hu WL *et al. (* 42)	review			Cancer Immunotherapy Based on Natural Killer Cells: Current Progress and New Opportunities	12.85
7	2020	Stadtmauer EA et al. (61)	Article			CRISPR-engineered T cells in patients with refractory cancer	12.03
8	2017	Rezvani K et al. (34)	review			Engineering Natural Killer Cells for Cancer Immunotherapy	11.93
9	2017	Zhang C et al. (20)	article	HER2	solid tumors	Chimeric Antigen Receptor-Engineered NK-92 Cells: An Off-the-Shelf Cellular Therapeutic for Targeted Elimination of Cancer Cells and Induction of Protective Antitumor Immunity	11.43
10	2017	Eyquem J et al. (62)	article			Targeting a CAR to the TRAC locus with CRISPR/Cas9 enhances tumour rejection	10.98
11	2016	Romee R et al. (63)	article		acute myeloid leukemia (AML)	Cytokine-induced memory-like natural killer cells exhibit enhanced responses against myeloid leukemia	10.84
12	2017	Rupp LJ et al. (64)	article			CRISPR/Cas9-mediated PD-1 disruption enhances anti-tumor efficacy of human chimeric antigen receptor T cells	10.3
13	2016	Guillerey C et al. (40)	review			Targeting natural killer cells in cancer immunotherapy	10.28
14	2016	Morvan MG et al. (65)	review			NK cells and cancer: you can teach innate cells new tricks	10.22

The articles ranked 7^th^ (strength: 12.03) (61), 10^th^ (strength: 10.98) (62) and 12^th^ (strength: 10.3) (64) in strength were all related research on CRISPR/Cas9 gene-editing technology in constructing engineered immune cells. The 11th-ranked article (strength: 10.84) published by Romee et al. (63) in 2016. Romee et al. proved that primary human NK cells could differentiate into memory-like NK cells after short-term pre-stimulation with IL-12, IL-15 and IL-18. In phase 1 clinical trial, these memory-like NK cells could proliferate in large numbers in AML patients, showing more vital anti-tumor ability.

By analyzing and summarizing the 9 articles (16, 20, 26–28, 61, 62, 64) that are in the state of citation burst, we can obtain the current emerging topics about the CAR-NK cell field:

- The basic research and clinical experimental research of CAR-NK cells in hematological malignancies involve targets including CD19 and CD33.
- The basic studies of CAR-NK cells in solid tumors involves tumors and targets, including ovarian cancer (mesothelin) and glioblastoma (HER2).
- Evaluating the efficacy and safety of CAR-NK cells in clinical trials.
- How to further improve the proliferation and persistence of NK cells *in vivo* (66). For example, promoting the differentiation of NK cells into memory-like NK cells.
- Optimizing and improving the CAR structure for NK cells to improve the anti-tumor ability of CAR-NK cells, such as taking NKG2D as transmembrane domain, and using costimulatory molecules (2B4, CD28, 4-1BB).
- The related research of CRISPR/Cas9 gene-editing technology in constructing engineered immune cells.

Combined with Knowledge Base, it can be found that the research of CAR-NK cells in hematological malignancies and solid tumors, and the evaluation of their efficacy and safety are the research focuses. In optimizing the structure of CAR, adding costimulatory molecules is an important strategy. Currently, CD28 and 4-1BB are commonly used. The combination of them can further enhance the anti-tumor effect of CAR-T cells (67). CD28 and 4-1BB can improve the function of CAR-T cells, which is also applicable to CAR-NK cells (68). In addition, researchers used NKG2D as transmembrane domain and added costimulatory molecule 2B4 to construct mesothelin-CAR-NK cells. The results showed that the anti-tumor effect of mesothelin-CAR-NK cells was similar to that of mesothelin-CAR-T cells (27). Interestingly, NKG2D can not only be used as a transmembrane domain of CAR, but also be used to construct single chain variable fragment (scFv) of CAR. Some studies showed that NKG2D-CAR-NK cells could effectively kill tumor cells expressing NKG2D ligands (69, 70). Furthermore, as a widely used gene editing technology, CRISPR/Cas9 technology is often applied to the construction of CAR-T cells and CAR-NK cells to enhance their anti-tumor effects (71, 72). Generally speaking, this section shows rich contents related to CAR-NK cells.

### Limitation

This study also has some limitations. First of all, all the data in this study came from the WoSCC database. Although the database contained most of the literature, there were still a few pieces of literature not included. Secondly, the quality of the included literature was uneven, which may lead to some degree of deviation in the analysis. Nevertheless, quantitative and visual analysis based on the literature data can help researchers quickly and intuitively understand the research trends, research directions, research hotspots and emerging topics in CAR-NK cell-related fields.

## Conclusion

CAR-NK cell therapy is a promising adoptive cell therapy (73). This study is the first time comprehensively and objectively analysing the CAR-NK cell field by combining bibliometrics with scientific knowledge maps. The number of publications related to CAR-NK cells was on the rise, especially from 2018 to 2021. In this field, the United States was in a leading position. The most active scientific research institution was Univ Texas, MD Anderson Cancer Center (USA). The journal with the most published papers was *Frontiers in immunology*, while the most co-cited was *Blood*. The researcher with the most published papers was Winfried S. Wels, while the most co-cited was Shannon L Maudeare. The research of CAR-NK cells in hematological malignancies and solid tumors (especially the selection of targets and the evaluation of efficacy and safety) was a research hotspot in this field. The emerging topics mainly included three aspects. First, further improve the proliferation and persistence of NK cells *in vivo*. Secondly, optimizing and improving the CAR structure for NK cells to improve the anti-tumor ability of CAR-NK cells. Thirdly, the related research of CRISPR/Cas9 gene-editing technology in constructing engineered immune cells.

In conclusion, this study provides a unique and objective perspective for the field of CAR-NK cells. We believe this study will provide a helpful reference for researchers interested in this field.

## Data availability statement

The original contributions presented in the study are included in the article/**Supplementary Material**. Further inquiries can be directed to the corresponding authors.

## Author contributions

JZ: Writing-original draft preparation, manuscript, investigation, and figure preparation. PC: Investigation, methodology, supervision. LM: Conceptualization, methodology, supervision, manuscript, figure preparation. All authors contributed to the article and approved the submitted version.

## Conflict of interest

The authors declare that the research was conducted in the absence of any commercial or financial relationships that could be construed as a potential conflict of interest.

## Publisher’s note

All claims expressed in this article are solely those of the authors and do not necessarily represent those of their affiliated organizations, or those of the publisher, the editors and the reviewers. Any product that may be evaluated in this article, or claim that may be made by its manufacturer, is not guaranteed or endorsed by the publisher.
