# Supplementary material for: A bibliometric and scientific knowledge-map study of the chimeric antigen receptor (CAR) natural killer (NK) cell-related research from 2010 to 2022
Source: Front Immunol. 2022 Aug 12;13:969196. doi: 10.3389/fimmu.2022.969196 (PMC9413055; doi:10.3389/fimmu.2022.969196)
Supplement: Supplementary file 1 [file DataSheet_1.pdf]

The retrieval formula of this study:

TS = (CAR-NK OR CAR-NK cell OR CAR NK cell OR CAR-engineered NK cell OR CAR-expressing NK cell OR CAR-transduced natural killer cell OR CAR-expressing natural killer cell OR chimeric antigen receptor natural killer-cell OR chimeric antigen receptor-expressing NK cell OR chimeric antigen receptor-transduced natural killer cell OR chimeric antigen receptor-engineered NK cell OR chimeric antigen receptor (CAR)-engineered NK cell OR chimeric antigen receptor (CAR)-transduced natural killer cell).

Inclusion criteria were as follows:
(1) timespan: January 1, 2010 to May 1, 2022;
(2) language: English;
(3) publication type: article or review.

Since the bibliometric analysis required collecting as much relevant literature as possible, the Exclusion criteria were set rather broadly，as follows:
(1) language: non-English;
(2) publication type: non-article or non-review.

These retrieved documents were exported in the form of “Full Record and Cited References” and saved as “Plain Text”. Finally, these files were named “download_*.txt”.
